# Communicating the Risk of MRSA: The Role of Clinical Practice, Regulation and Other Policies in Five European Countries

**DOI:** 10.3389/fpubh.2017.00044

**Published:** 2017-03-17

**Authors:** Petra Dickmann, Sam Keeping, Nora Döring, Andrea E. Schmidt, Claudia Binder, Sergio Ariño-Blasco, Joan Gil

**Affiliations:** ^1^London School of Economics and Political Science (LSE), LSE Health, London, UK; ^2^dickmann risk communication (drc), London, UK; ^3^Department for Anaesthesiology and Critical Care Medicine, Jena University Hospital, Jena, Germany; ^4^Department of Health Services Research, School for Public Health and Primary Care (Caphri) of the Faculty of Health, Medicine and Life Sciences, Maastricht University, Maastricht, Netherlands; ^5^Department of Public Health Sciences, Karolinska Institutet, Stockholm, Sweden; ^6^Austrian Public Health Institute Ltd., Vienna, Austria; ^7^European Centre for Social Welfare Policy and Research, Vienna, Austria; ^8^Universitat Internacional de Catalunya Hospital General Granollers, Granollers, Spain; ^9^Department of Economics and BEAT Research Institute, University of Barcelona, Barcelona, Spain

**Keywords:** meticillin-resistant *Staphylococcus aureus*, health-care-associated infections, risk communication, infection control, health policy

## Abstract

**Background:**

The threat posed by Meticillin-resistant *Staphylococcus aureus* (MRSA) has taken on an increasingly pan-European dimension. This article aims to provide an overview of the different approaches to the control of MRSA adopted in five European countries (Austria, Germany, Netherlands, Spain, and the UK) and discusses data and reporting mechanisms, regulations, guidelines, and health policy approaches with a focus on risk communication. Our hypothesis is that current infection control practices in different European countries are implicit messages that contribute to the health-related risk communication and subsequently to the public perception of risk posed by MRSA. A reporting template was used to systematically collect information from each country.

**Discussion:**

Large variation in approaches was observed between countries. However, there were a number of consistent themes relevant to the communication of key information regarding MRSA, including misleading messages, inconsistencies in content and application of published guidelines, and frictions between the official communication and their adoption on provider level.

**Summary:**

The variability of recommendations within, and across, countries could be contributing to the perception of inconsistency. Having inconsistent guidelines and practices in place may also be affecting the level at which recommended behaviors are adopted. The discrepancy between the official, explicit health messages around MRSA and the implicit messages stemming from the performance of infection control measures should, therefore, be a key target for those wishing to improve risk communication.

## Introduction

Health-care-associated infections (HCAIs) place a significant burden on health systems in terms of both morbidity and mortality, and their effects are felt far beyond just those utilizing health-care services (1). An already serious situation is now being exacerbated by the growth of antibiotic-resistant bacteria, such as meticillin-resistant *Staphylococcus aureus* (MRSA). MRSA is a bacterium which is resistant to β-lactam antibiotics, a group of antibiotic treatments that includes penicillin and cephalosporin. Infection with MRSA can lead to a variety of sequelae, including ventilator-associated pneumonia, chronic wound infection, bloodstream infection (bacteremia), and septic conditions, which in some circumstances can lead to death.

The prevalence of MRSA varies across Europe, with a general trend of increasing prevalence from north to the south (2–4). The reasons for these observed differences are not yet fully understood, although variation in prevention and control strategies (5), design of health-care facilities (6), staff to patient ratios (7), patterns of antibiotic usage (8), and the implementation of antibiotic stewardship (9, 10) are thought to be contributing factors. As travel between European states becomes more common, with citizens free, within certain limits, to pursue elective treatment in a European country other than their own, the scope for transmission of infectious pathogens to areas where they are not endemic becomes ever greater (11, 12). Therefore, cross-border regions now face a particular challenge in coping with patients coming from neighboring countries where different regulations and practices are in place to prevent, detect, and respond to infectious agents (13). The rise of MRSA and the European cross border health-care legislation to allow treatment in another EU country have led to the formation of a number of MRSA networks, founded on bilateral agreements between countries with shared borders about how best to manage the issues outlined above (14).

Increases in the burden associated with HCAIs and the growth of antimicrobial resistance have led to heightened awareness both within the lay population and among politicians (15). The Chief Medical Officer in the UK has even gone so far as to place the threat posed by antimicrobial resistant on a par with that of terrorism (16). The European Centre for Disease Prevention and Control (ECDC) has placed the “Antimicrobial Resistance and Healthcare-Associated Infections Programme” among its top priorities for the future (17). The ECDC has also stated that a major part of the problem stems from deficiencies in the way health-related issues are communicated, and has encouraged EU member states to improve their risk communication strategies (18).

Risk communication is a wide and multi-faceted field, and is considered an important approach in the fight against the spread of infectious diseases through the impact it can have on the adoption of appropriate behaviors, e.g., frequent hand washing to limit carriage and infection (19). Recent research has highlighted a number of areas which are key to understanding effective risk communication, such as the nature and quality of information provided to patients and health-care workers (20), patients’ and the general public’s perceived information needs (21, 22), and role of the media (23). However, little research has been carried out into the impact of institutional arrangements on specific communication strategies (24).

We conceptualize risk communication as not simply explicit communications but also the implicit messages of institutional arrangements (differences in policies, inconsistencies of implementation, etc.). In order to test this assumption, we examined the implementation of various MRSA policies across five European countries for any evidence that environmental factors contribute as implicit and “autonomous” risk communication next to traditional explicit and “voluntary” forms.

The article begins by discussing the situation in each of the countries with regard to the following areas, while reflecting on the underlying epidemiological rationale for each:

- data and reporting mechanisms,
- regulations and guidelines,
- health policy approaches with a focus on risk communication, and
- implicit messages of current practices.

In the second section, we move on to discuss how different national strategies to contain MRSA infection are implicit “messages” and contribute to explicit risk communication strategies.

## Materials and Methods

We collected information on the situation with regard to MRSA in five European countries until 2011: Austria, Germany, Netherlands, Spain, and the UK. These countries were chosen as they represent varying prevalence, response strategies, overall health system organization and modes of communication.

A template was designed to systematically collect information about five areas: (1) data collection and reporting mechanisms; (2) the regulatory framework; (3) clinical guideline design; (4) implementation of guidelines; and (5) other relevant health policy measures. The intended and unintended effects of policies in each of these categories were then compared to explicit communication strategies first by country and then across countries. Information was drawn from scientific and gray literature and complemented, where necessary, by stakeholder interviews.

To help interpret the results and how the various measures compare to one another, we developed a stepwise classification for different stages of MRSA (see Table 1).

**Table 1:** **Classification of meticillin-resistant *Staphylococcus aureus* (MRSA) from an infection control perspective**.

Level I	Colonization	MRSA can be found on the skin, in the nasal cavity, or in a wound. The colonization itself is not an ostensible health problem; however, it can lead to an acute infection and most importantly MRSA is contagious from the level of colonization on. Healthy people are still healthy with MRSA colonization, but could spread the bacterium to others. For ill people, MRSA colonization could lead to an infection with the bacterium. With an easy and non-invasive swab the colonization can be identified. A proven colonization can be sanitized with antibiotic cream and antiseptic washings
Level II	Infection	MRSA is on the skin or in the nasal cavities or in wounds and causes a reaction in the immune system, e.g., inflammation, antibodies, fever, etc. The infection can be proven by a blood test showing the systemic signs of an immune system reaction, and the bacterium can be found locally in the infected area. Patients with a compromised immune system or with skin problem are more susceptible to develop MRSA infections than healthy people
Level III	Bacteremia	Bacteria can be found in the bloodstream. In combination with clinical symptoms, this is called sepsis. A septic condition is a serious, life threatening medical condition. Bacteremia is proven by a blood sample where bacteria can be found. A blood test for bacteremia is only performed when medically indicated, e.g., a patient developing a septic clinical condition

## Results

We present narratively a summary of results; the details can be found in Table 2.

**Table 2:** **Overview of results in five EU countries**.

	Austria	Germany	Netherlands	Spain (Catalonia—autonomous community)	United Kingdom
*Reporting situation: mandatory reporting* (frequency; level I = colonization, level II = infection, level III = bacteremia), *Register*	*No mandatory reporting* – Voluntary basis for hospitals (level III) – Annual reporting of routine laboratory data (level III) – Only one federal states has a registry	*Mandatory*: quarterly reporting of bacteremia (level III) to the Robert Koch Institute (RKI) (since 07/2009) – Voluntary: Point prevalence information of infections (level II) – No information on colonization (level I)	*Mandatory* reporting of level I-III – Samples of bacteria strains (level I and II) sent to RIVM for classification	*No mandatory* reporting – Unified surveillance program (VINCat program): annual collection of MRSA ratio, incidence and bacteremia (level III) information	*Mandatory* weekly reporting of bacteremia (level III) since April 2004 – Mandatory reporting of MRSA mortality
*Regulatory bodies, name, role*: issue guidelines; control implementation?, *impact*	*Federal approach* – There is a basic national legal framework^a^, but each of the nine federal states has their own laws. – Each Hospital develops its own infection control plan	*Federal approach with national guidance* – RKI on national level. Health is in the responsibility of the 16 federal states, thus a commission has been established at the RKI representing the federal states (*Commission for Hospital hygiene and Preventions of Infections*/KRINKO). – German Society for Hospital Hygiene advises KRINKO – While KRINKO releases recommendations for the federal states, the German Protection Infection Act (IfSG) provides the legal framework for infection control in Germany on national level	*National approach* – Ministry of Health, Welfare, and Sports – Netherlands Institute for public health and the environment (RIVM) – Health Council of the Netherland (GR) – The health care inspectorate (IGZ) – The Dutch working party on infection prevention (WIP)	*Regional approach* – Public Health Directorate in the Public Health Agency – Hospital networks have established nosocomial infection control program (VINCat) – All regulations apply for regional level	*National approach* – *England*: Care Quality Commission (CQC) as laid down by the Health and Social Care Act 2008; Foundation trusts have their own independent regulator known as Monitor – *Scotland*: Social Care and Social Work Improvement Scotland and Healthcare Improvement Scotland – *Wales*: Health Inspectorate for Wales, created in 2004, and the Care and Social Services Inspectorate for Wales – *NI*: Regulation and Quality Improvement Authority
*Guidelines*	*Guidelines*: each hospital has its own guideline but all refer to the recommendations by Institute of Microbiology of Universities	*Guidelines*: German antibiotic resistance strategy (DART) KRINKO/RKI recommendations (Versions: 1999, 2005, 2011)	*Guidelines*: “Search and destroy” since 1986	*Guidelines*: consensus document: unify treatment of MRSA infection by a set of evidence-based recommendations	*Guidelines*: the Health and Social Care Act (2008) Code of Practice for the NHS on the prevention and control of health-care-associated infections (HCAIs): risk of infection posed to patients is kept to a minimum
*Basic principles*	*Principle* – Risk-based testing of known or highly likely patients – Isolation after positive test results	*Principle*: risk-based testing of known or highly likely MRSA patients – Isolation when tested positive until three negative swaps – Barrier nursing for HCW – Interventions in MRSA patients should be restricted to those deemed absolutely necessary – No routine screening for patients or staff	*PrincipleSearch*: Active screening of patients and staff. *destroy*: decolonization and treatment based on guidelines developed by Dutch Working Party on Antibiotic Policy (SWAB) – Isolation based on risk assessment from first contact on	*Principle* – Risk-based testing (nasal swabs) – Isolation for positive and waiting for results patients	*Principle* – 10 registration criteria for the prevention and control, such as information, clean environment, identification of infected patients, isolation facilities – As of 2008 screening of high-risk cohorts, in particular A&E admissions and pre-operative surgical assessment patients – As of April 2009 all elective admissions must be screened for MRSA
*Implementation, routine, and consequences*	*Legal obligation* for hospitals to implement measure to assure hygiene quality – Federal states carry out regular checks (differ among federal states)	*Legal obligation* for hospitals to implement measure to assure hygiene quality – Implementation has not been routinely controlled yet	*Legal obligation* for hospitals to implement measure to assure hygiene quality. – Implementation controlled by the Health Care Inspectorate	*Legal*: Infectious diseases committees are mandatory in the public network of hospitals in Catalonia – Implementation controlled by The Catalonian Health Department, throughout periodic accreditation processes	*Legal obligation*: Deadline for implementation no later than 2011 – Implementation assessed by the CQC
*Health policy, national action plan, regional networks, and antibiotic stewardship*	– Quality Committee to contribute to the development of quality management – Networks: National Reference Centre for Nosocomial Infections and Antibiotics Resistance – National Action Plan and a National Antimicrobial Strategy is currently being developed – Antibiotic StewardshipEuropean Networks	– New Infection Protection Act regarding nosocomial infections (9 June 2011). The law is mandatory for all 16 Länder and the changes have to be implemented by March 31, 2012 **Key points:** – *Hygiene Commission ART*: a commission for “anti-infectiva resistance and therapy” (ART) is to be established with diagnostic and treatment list – *Joint Federal Committee* “Gemeinsamer Bundesausschuss” releases guidelines to hygiene and quality management – Sanitation: the outpatient sanitation of MRSA patients should be reimbursed better for GPs to motivate them to follow up the treatment of MRSA outpatients. – Strong hospital network especially in cross-border regions, e.g., *EUREGIO* Twente/Münsterland	The Dutch Working Party on Infection Prevention (WIP)*EUREGIO* Twente-Muensterland MRSA network*EurSafety Health Net*, MRSA networks for the Euroegio Meuse Rhine, Rhine-Meuse-Nord, Rhine-Waal, Gronau-Enschede, Ems-Dollert regio	Some quality indicators (prevalence of nosocomial infection) are incorporated into incentive policies among professionals as part of the “*Management by Objectives*” approach. Actors involved in MRSA policies are: local infection control committees, infection control nurse, consultant in infectious diseases, microbiologist, and clinical staff	Health Protection Agency – collects routine surveillance data on infection rates, provides training and specialist advice on ways to deal with infections – acts as a conduit for the sharing of information between providers – works with the general public to ensure key information on the threats posed by infections is easily accessible
*Explicit communication*, information policy, and key messages	– Each hospital creates their own guidelines, adapted to their specific circumstances and based on the hygiene regulations enshrined by law and the recommendations published by the responsible scientific institutions at universities – Dissemination of information for patients and visitors in an *ad hoc* way – In general, little media attention regarding MRSA	KRINKO guidelines point out that information and communication is key to successfully respond to the health threat posed by MRSA – Information and communication needs have not been investigated or recommended in the new guidelines	– Information is available at hospitals and nursing homes – Information for the general public, hospital staff and policy makers about MRSA is available through the MRSA network described above	– Information available for health professionals, patients and general public	– Two of the 10 criteria in the Code of Practice for the NHS on the prevention and control of HCAIs relate specifically to the provision of information – Despite the laws and effort surrounding improving information the results have been mixed

*^a^Hospitals and medical institutions Act/Krankenanstalten- und Kuranstaltengesetz*.

### Data Situation

Of the five countries, three countries have mandatory reporting (Germany, Netherlands, and UK), while others (Austria and Spain) report on a voluntary basis. Only the Netherlands report MRSA from colonization level onward; the majority of countries (Austria, Germany, Spain, UK) only report bacteremia (level III), which is the most serious consequence of infection and only present in a minority of cases. The UK, however, also screen for MRSA status prior to elective surgery, but this information is not officially reported. There is no consistent information about the “contagious burden,” meaning the provision of information about colonization and infections, from level I on where MRSA is contagious and can be passed to others (25).

### Regulatory Bodies

Regulatory bodies are built based on the political structure of the country where they are based. In Austria, Germany, and Spain health-care provision and management is in the responsibility of federal states or autonomous communities. The Netherlands and the UK follow a national approach.

### Guidelines

The Netherlands has opted to pursue an active screening policy (“search and destroy”). Risk-based testing is in place in Austria, Germany, Spain, and the UK, with the UK requiring screening of elective admissions to hospitals. Isolation practice for positive cases is basically the same in the five countries. However, the time lag between entering the hospital and being identified as MRSA carrier is critical as a variety of admission procedures, examinations, and clinical investigations at the beginning of a hospital stay increase the likelihood to spread infectious diseases. The Netherlands, again, stand out by placing patients with an unknown MRSA status in isolation rooms; Spain places patients awaiting their results in isolation as well as, whenever possible (individual room availability), those who are previous carriers or high-risk patients (in one autonomous community, Catalonia).

Health-care workers in the four countries (Austria, Germany, Spain, UK) are not screened on a regular basis, meaning that those who are a high-risk group in every countries’ guidelines and work where the contracting and spread of infectious disease occurs most readily are unaware of their own MRSA status (26). The Netherlands screen staff regularly.

### Implementation

All countries investigated have put legal obligations in place to implement their guidelines. However, only sporadic, if any, verifications and checks are being carried out.

### Health Policy

All the countries under investigation have increased their awareness for MRSA and have developed national action plans, regional and international networks of hospitals and laboratories, antibiotic stewardship, strengthened their legislation, and created incentives to reduce the prevalence of MRSA (9).

### Communication

Information and knowledge play a crucial role in the management of MRSA, and European health policy also puts special attention on communication. All five countries thus prioritize the provision of information to various target groups (health professionals, patients, and general public).

## Discussion

We first comment the results of the five countries, then discuss the epidemiological rationale and the implication for risk communication.

### Situation in the Five Countries

#### Reporting

Comprehensive data on prevalence is difficult to obtain as most mandatory and voluntary surveillance systems were found to only cover MRSA bacteremia. Point prevalence studies and surveys that have attempted to capture the situation have also been based on information provided by hospitals on a voluntary basis. This has contributed to the sporadic nature of evidence around MRSA, and thus, current data do not appropriately reflect the ubiquitous nature of the treat of infection (3).

Moving the reporting downstream with the reporting of bacteremia, level III, is posited to be one way to keep the absolute numbers down. In regard to communication, this contributes to keeping perception of infection rates artificially low. This could be appealing to policy makers as the small numbers reported cause less concern than if the prevalence of infection were to be revealed (27–29). It is, however, only an artifact of the reporting and not reflective of the underlying epidemiology of the infectious disease.

#### Regulatory Bodies

Having different regulatory bodies could possibly lead to variability in terms of guidelines and recommendations and their implementation. The variability of recommendations within and across countries could contribute to the perception of inconsistency.

#### Guidelines

The rigor of infection control is not appropriately reflected in the guidelines: MRSA patients are constantly contagious and not only after identification; health-care workers fall under the risk groups for screening in every country, but are, with the exception of the Netherlands, not screened.

#### Implementation

Concerns about the implementation of the guidelines are frequently raised. Surveys from Germany and the UK support these findings. The authors showed that a minority of the hospitals have consistently implemented guidelines (30, 31).

#### Health Policy

The consistent adoption of policy in the guidelines and the implementation into the daily practice is, however, subject to discussion. The gap between the policy and its implementation is not just a medical problem but also influences the perception of health risks and could affect compliance with the intended behavior (32).

#### Communication

The explicit information strategy is only one aspect of communication (“voluntary communication”); another aspect is how messages that are expressed in the official statements are “executed” on the ground of the daily reality of health care (“autonomous communication”). Infection control measures, their implementation into the medical environment, and their frictions are tacit acts of communication yet little attention has been spent on how to monitor and improve the way they contribute to the conveyance of key health messages.

### Epidemiological Rationale

#### Infection Control Approach

There are, generally speaking, two basic strategies for responding to infectious diseases that emerge from the analysis: a “specific” and a “general” approach.

The specific approach relies on the identification of those patients already infected with MRSA. Once an MRSA patient is identified and known (“red flagged”), appropriate measures can be taken. These measures range from sanitation of skin colonization and/or treatment of the infection. The patient should also be isolated from the hospital environment in order to prevent the transmission of the pathogen. Once an MRSA patient has been identified, health-care workers have to wear personal protection equipment and apply stringent hygiene measures. The MRSA patient’s room is often labeled with a sign signaling the contagious status. There is a general consensus regarding isolation and hygiene practices. However, the crucial choice of strategy for identification in the specific approach is controversial. Only the Netherlands has a pro-active screening policy; all the other countries studied used a process of reactive screening based on risk assessment, with the UK also requiring pre-hospital screening for elective admissions. Hospitals across the four “reactive” EU countries (Austria, Germany, Spain, UK) appear very reluctant to screen patients for a number of reasons. One is that the care for and treatment of MRSA patients places greater demands on the attending nurses and clinicians, meaning that there could be an incentive to avoid correct MRSA classification. Additional measures, such as spatial requirements (single isolation rooms), differential treatment guidelines, and consequences for staff and ward routines, aggravate the situation. Also, at the hospital level, it ostensibly requires more time and resources to care for MRSA patients and, therefore, those facing budgetary pressure may possibly be more inclined to avoid diagnosis, despite the fact that the costs of cases progressing to bacteremia may outweigh the costs of an active screening policy (33–37).

If contagious patients are not identified, a “general” approach with stringent measures to guarantee good hygiene has to be adhered to by all health-care workers, patients, and visitors. This system of infection control includes, among others, requirements such as strict hand hygiene, regular cleaning, and disinfection of surfaces. To promote the general hygiene approach, good communication is crucial to ensure that everyone is aware of what they need to do in order to avoid infection and stop further spread. Frictions between infection management and communication could affect the adoption of the recommended behavior.

#### Ethical Problems

The handling for MRSA patients raises some ethical questions regarding whether there is equal treatment of isolated and contagious patients. The restriction of physical transport and transfer forms part of the infection control recommendations in the European countries investigated. However, it is an increasingly controversial and sensitive aspect of the prescribed treatment of MRSA patients as many feel it could compromise the quality of clinical care (38). Most guidelines recommend that some invasive interventions be confined to the room used by MRSA patients (39). In the case where interventions performed in the patient’s room would be better carried out elsewhere, the lack of an optimal environment could lead to reduced performance on the part of clinicians. Infectious patients are put at the end of the day’s surgery schedule and are more likely to be postponed due to emergencies in the surgical program. The avoidance of invasive diagnostics alongside being the “last operation on the schedule” could comprise the medical treatment of an often critically ill patient.

#### Organizational Aspects

Meticillin-resistant *Staphylococcus aureus* is also an occupational health problem. Health-care workers are not routinely screened for MRSA colonization or infection—apart from in the Netherlands. This reluctance to identify infectious staff could be seen in the context of health-care organization. If a staff member is colonized with MRSA, they are not allowed to work in their usual locations. This poses a burden to the workplace organization in terms of the potential for inconsistent labor supply, in particular in an already overstretched working environment. Hospitals and countries in which the intensive care unit carer/nurse to patient ratio is 1:1 report basically no problems of nosocomial infections (40). This adds weight to the hypothesis that a major contributor to increasing prevalence of MRSA is the high patient–health-care worker ratio (41).

#### Architecture

The guidelines also point out the importance of spatial distancing and the role of architecture—a challenge which has not been met by modern hospitals and it remains unclear whether this concern regarding the spread of infectious disease will be met in the future (6).

### Risk Communication Strategy

The “general” approach, i.e., the basic tools for fighting an infectious disease epidemic, all features prominently in all national guidelines: knowledge, training, information, networks, and collaboration. But the question remains of how well are the recommendations implemented at the provider levels especially in terms of their risk communication practices.

#### Conflicting Messages

From a patient perspective, the discharge of MRSA patients is an ostensibly incongruent routine. After being treated in strict isolation and under a stringent hygiene regimen, patients are simply discharged and informed that MRSA is no risk for healthy people (42). This dissonance could lead to confusion and distress for patients, relatives, and visitors and add further to potential misperceptions of their health risk to others.

#### Knowledge

Despite the laws and efforts surrounding improving information and communication, knowledge levels have been mixed. The lack of knowledge in health-care professionals is seen as an influencing factor for increasing prevalence of nosocomial infections (43–46).

#### Risk Communication Policy

The risk communication policies of the five EU countries were found to contain only information relating to risks faced by people directly involved in health care, such as hospital staff and patients. These policies were based on the same risk assessment that is used to determine which patients should be screened for MRSA. It focuses on health-care workers, long-term care, chronically ill patients, and patients facing surgery. There has only been little effort to address MRSA as problem in and for the general population (47). This narrow view is congruent with the lack of concern for the role of other factors, such as behavior, implementation of guidelines, etc., seen in other areas.

#### Media Coverage

The consensus in the literature is that the UK media coverage, especially within the tabloid newspapers, has been at times sensationalist. However, the pressure placed on governments in response to the extensive media coverage has played a significant role in a number of policy changes which have contributed to the decline in incidence of deaths and bacteremia associated with MRSA (48, 49).

#### Public Perception

The infection threat posed by MRSA is difficult to communicate. Researchers have blamed contradicting risk communication about necessary hygienic measures as one problem (50). They see that even necessary cleaning routines have not been implemented in hygiene plans (50). More importantly, risk communication messages might have influenced the risk perception of health-care workers, patients, and the public inappropriately. The message that MRSA is not an infectious diseases agent that can lead to outbreaks outside of health-care facilities and also does not do harm to family members has direct consequences for the epidemiology of MRSA and could, in turn, have led to the perception that MRSA is a harmless pathogen only affecting those who are already ill (42).

### Summary

The data situation in the countries is patchy at best, and thus it is difficult to offer up any firm conclusions regarding the overall burden of disease in the countries studied. What is clear, however, is that knowledge and information about the infectious disease burden is limited in the general population, and this lack of clarity has led to a growth in misconceptions surrounding the threat of MRSA.

The variability of recommendations within, and across, countries could be contributing to the perception of inconsistency, and therefore, this should be looked, potentially, as part of a wider strategy designed to improve risk communication. Having inconsistent guidelines and practices in place may also be affecting the level at which recommended behaviors are adopted. This is an area that would require more research.

The risk communication of MRSA has several weaknesses: there are misleading messages in the official statements and a gap between the official communications and guidelines, with inconsistent adoption of the latter at the provider level.

This discrepancy between the official, explicit health messages around MRSA and the implicit messages stemming from the performance of infection control measures should, therefore, be a key target for those wishing to improve the accuracy of perceptions regarding the health risks of MRSA.

### Recommendation

The increasing burden of antimicrobial resistance and health-care-associated infections has been reflected in growing public awareness, for example with major health policy organizations urging countries to improve their risk communication and MRSA prevention strategies. These ought to be revised to also address the general public. Thus far, most countries have adopted a universal risk-based approach addressing affected groups, without differentiating between groups, or addressing the wider public. Health policy and practice has also focused on individual infection control measures and place the majority of responsibility on the individual. Organizational aspects (patient/health-care worker ratios, architecture, etc.) have not been prominently discussed. The rational use of antibiotics and antibiotic stewardship is a significant move designed to place greater responsibility for the control of HCAIs on medical professionals. However, the key message, often included in national risk communication strategies, that MRSA is a problem only for those who are already ill is misleading. In fact, MRSA is a problem affecting society as a whole. MRSA is a problem for healthy people as they can transmit the disease; MRSA is a problem for treating too many patients in too narrow spaces. MRSA is a problem because MRSA patients are only reluctantly identified. And finally, MRSA is a problem because the explicit and implicit messages of MRSA are often inconsistent, if not contradictory.

The problem of health care-related infections and antimicrobial resistance can only be tackled in a more holistic approach regarding reconsideration of affected groups, health-care organization, architecture, and a rational use of antibiotics—and by revising a risk communication strategy accordingly.

## Data Availability

All data used are provided in the tables.

## Ethics Statement

No ethics approval or consents from patients or participants were sought as this is an analysis of published literature and health policies. No confidential information was disclosed.

## Author Contributions

PD developed the concept, provided the information from Germany, drafted the manuscript, and oversaw the project. SK provided information from the UK and contributed to the discussion and the manuscript writing; ND provided information from the Netherlands and contributed to the discussion and the manuscript writing; AS and CP provided information from Austria and contributed to the discussion and the manuscript writing; and SA-B and JG provided information from Spain and contributed to the discussion and the manuscript writing. All authors approve the final version of the manuscript.

## Conflict of Interest Statement

The authors declare that the research was conducted in the absence of any commercial or financial relationships that could be construed as a potential conflict of interest.
